# Role of redox-active axial ligands of metal porphyrins adsorbed at solid–liquid interfaces in a liquid-STM setup

**DOI:** 10.3762/bjnano.11.110

**Published:** 2020-08-24

**Authors:** Thomas Habets, Sylvia Speller, Johannes A A W Elemans

**Affiliations:** 1 Radboud University, Institute for Molecules and Materials (IMM), 6525 AJ Nijmegen, Netherlands; 2 University of Rostock, Institute of Physics, Albert-Einstein-Straße 23, 18059 Rostock, Germany; 3 University of Rostock, Department Life, Light, Matter, Albert-Einstein-Straße 25, 18059 Rostock, Germany

**Keywords:** manganese, porphyrins, redox reactions, scanning tunneling microscopy, solid–liquid interface

## Abstract

In a liquid-STM setup environment, the redox behavior of manganese porphyrins was studied at various solid–liquid interfaces. In the presence of a solution of Mn(III)Cl porphyrins in 1-phenyloctane, which was placed at a conductive surface, large and constant additional currents relative to a set tunneling current were observed, which varied with the magnitude of the applied bias voltage. These currents occurred regardless of the type of surface (HOPG or Au(111)) or tip material (PtIr, Au or W). The additional currents were ascribed to the occurrence of redox reactions in which chloride is oxidized to chlorine and the Mn(III) center of the porphyrin moiety is reduced to Mn(II). The resulting Mn(II) porphyrin products were identified by UV–vis analysis of the liquid phase. For solutions of Mn(III) porphyrins with non-redox active acetate instead of chloride axial ligands, the currents remained absent.

## Introduction

Manganese(III) porphyrins are well-known catalysts for the epoxidation of alkenes [1–4]. The manganese center of the porphyrin moiety serves as a coordination site for an oxygen atom, which subsequently is inserted into the double bond of the alkene. A variety of oxygen donors can be used to oxidize the manganese center to an active manganese-oxo (Mn=O) species, such as iodosylbenzene, hypochlorite and hydrogen peroxide. The use of the environmentally most benign oxidant, molecular oxygen (O_2_), is also possible, but comes with a drawback. To be able to generate an Mn=O complex, the Mn(III) porphyrin first needs to be reduced to a Mn(II) porphyrin, which can subsequently coordinate to O_2_ and activate it for splitting of the O–O bond. To accomplish this, generally a co-reductor (e.g., isobutyric aldehyde [3,5–6]) is included in the catalytic system in order to facilitate the first reduction step.

**Figure 1:**
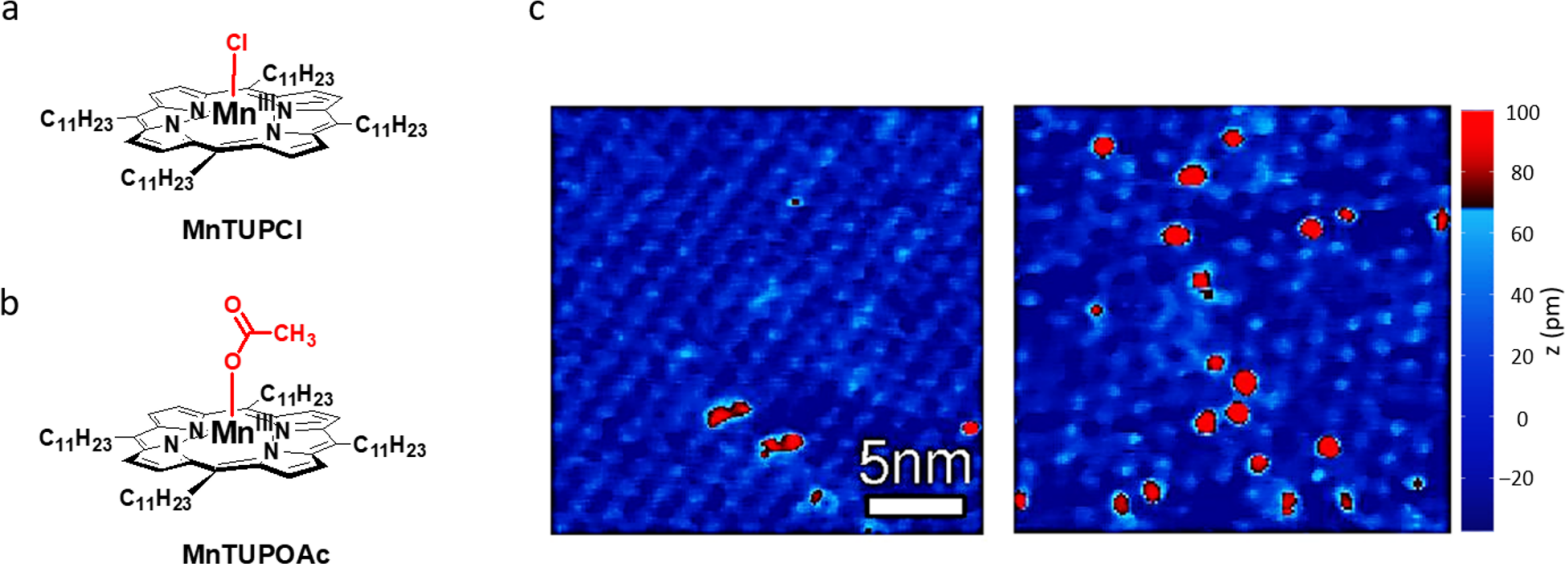
(a) Molecular structure of **MnTUPCl**. (b) Molecular structure of **MnTUPOAc**. (c) STM images of a monolayer of **MnTUPCl** at a Au(111)–*n*-tetradecane interface; the light blue spots are the native **MnTUPCl** molecules, the red spots their respective Mn=O complexes; left image: in argon atmosphere, a few Mn=O species are present; right image: in oxygen atmosphere, more Mn=O species have been formed. The color bar on the right indicates apparent heights. The STM images were adapted from our previous work [7].

Previously, our group has investigated the catalytic properties of manganese porphyrins at the single-molecule level, employing scanning tunneling microscopy (STM) [7–9]. Since our aim was to stay as close as possible to the laboratory conditions at which catalysis takes place (typically in an organic solvent under ambient conditions), we carried out our STM studies at a solid–liquid interface at room temperature. We found that while the porphyrin catalyst **MnTUPCl** (tetrakis-*meso*-undecylporphyrin manganese(III) chloride, Figure 1a) is fully inert in *n*-tetradecane solution, it becomes catalytically active in the epoxidation of alkenes when it is adsorbed at the interface of a Au(111) substrate and an *n*-tetradecane solution of the compound [7]. From real-time topographic signature changes in the STM images (Figure 1c), combined with optical reflectance spectroscopy, it was concluded that Mn=O complexes were readily formed in the presence of O_2_ gas. This was a surprising result, since a chemical co-reductor was absent. It turned out that a sufficiently negatively biased surface was responsible for the reduction of the metal centers of the porphyrin moieties from Mn(III) to Mn(II), which activated them to coordinate and dissociate O_2_.

Although these STM studies provided fundamentally new insights into reactivity at the single-molecule scale, some mechanistic aspects still remained unclear. For instance, the reduction of the Mn(III) porphyrin to a Mn(II) porphyrin also involves the dissociation of the axially coordinating Cl ligand from the metal center, and it is as yet unclear what the fate of this ligand is. In the catalysis study with **MnTUPCl** at the Au(111) surface, two possible mechanisms were proposed [7]: (i) A surface gold atom coordinates to the manganese center of **MnTUPCl** in an axial ligand-like fashion, inducing a chlorine radical to dissociate, thereby reducing the Mn(III) center to Mn(II), or (ii) the surface actively reduces **MnTUPCl** by donating an electron, followed by dissociation of a chloride anion.

In this paper, we investigate in more detail the role of the axially coordinating Cl ligand of **MnTUPCl** at a solid–liquid interface in a liquid-STM setup. By systematically varying the experimental conditions in terms of type of substrate, solvent, solute, and concentration of the solute, we will demonstrate that the ligand can in fact be involved in redox processes in the liquid-STM setup.

## Results and Discussion

One of the possible mechanisms for axial ligand dissociation mentioned in the introduction was the direct attachment of a gold atom to the manganese center of **MnTUPCl**, coming from the Au(111) surface below. As a first experiment we therefore decided to also investigate with STM the behavior of **MnTUPCl** at a HOPG surface instead of a Au(111) surface, since in that case no direct metal atom coordination from the substrate is possible. To our surprise we found a strong influence of the solvent on the success of imaging the molecules with STM. When *n*-tetradecane was used as the solvent, monolayers of **MnTUPCl** readily formed on Au(111) (Figure 1c), while poorly organized and dynamic layers were formed on HOPG (not shown). When, however, 1-phenyloctane, a broadly applied aromatic solvent in liquid-STM studies, was used, it was impossible to image the surface or adsorbed molecules regardless of the used surface, due to the occurrence of a large additional increase in measured tunneling current. While the imaging of monolayers of **MnTUPCl** and other metal porphyrins based on the TUP-ligand generally requires a tunneling set current of less than 5 pA [7,10–13], now additional currents of several tens of picoamperes were present. Remarkably, the additional currents were never observed in experiments with analogous porphyrins with a copper(II) or cobalt(II) center [10–11]. In the following, we will investigate the redox behavior of **MnTUPCl** at the solid–liquid interface in more detail.

It is well-established that during and after the addition of solutions of molecules to the sample surface in an STM setup, the observed current between the tip and sample can increase. This increase may, for example, be the result of a mechanical disturbance made to the system upon adding the liquid, the presence of ions in the solution, and/or polarization of the solution or solutes therein. In most cases the additional current is of the order of the noise level of the STM (approx. 1 pA), or at least significantly smaller than the applied tunneling current. However, after the addition of a 1-phenyloctane solution of **MnTUPCl** (*c* = 1 × 10^−4^ M), significant currents with a magnitude of about 50 pA were measured between the Pt_90_Ir_10_ tip and the HOPG sample. The magnitude of the additional current increased with an increasing concentration of the compound, or with an increasing magnitude of the bias voltage. Over a time of minutes to hours, the additional current decayed somewhat, but not to zero. In order to qualify the behavior of the additional current, we slightly adjusted our STM setup. Conventionally, we added with a syringe a small droplet (5–10 µL) of solution to a sample of HOPG, which was contained around the tip and at the surface because of the surface tension of the droplet. In our modified setup, we used a liquid cell to contain a larger and well-defined volume of solution at the sample surface, ensuring that the concentration of the solutes remains stable during the sometimes prolonged measurements. During the experiments the voltage difference between the tip and sample surface was varied using the STM controller, while the current and the applied voltage were recorded using a data logger. The quantitative behavior of solutions of **MnTUPCl** at the surface of the STM setup did not change by using this modification, in the sense that the magnitude of the current still increased with the concentration and the magnitude of the bias voltage. After the addition of 350 µL of a 1-phenyloctane solution of **MnTUPCl** (*c* = 1 x 10^−4^ M) to the modified STM setup, currents of 40 ± 20 pA were measured between the tip and the HOPG sample (at a bias voltage of 0 mV). This current remained even when the tip was manually retracted far out of tunneling range (tip sample distance: 12 ± 2 µm), while still staying in contact with the solution.

**Figure 2:**
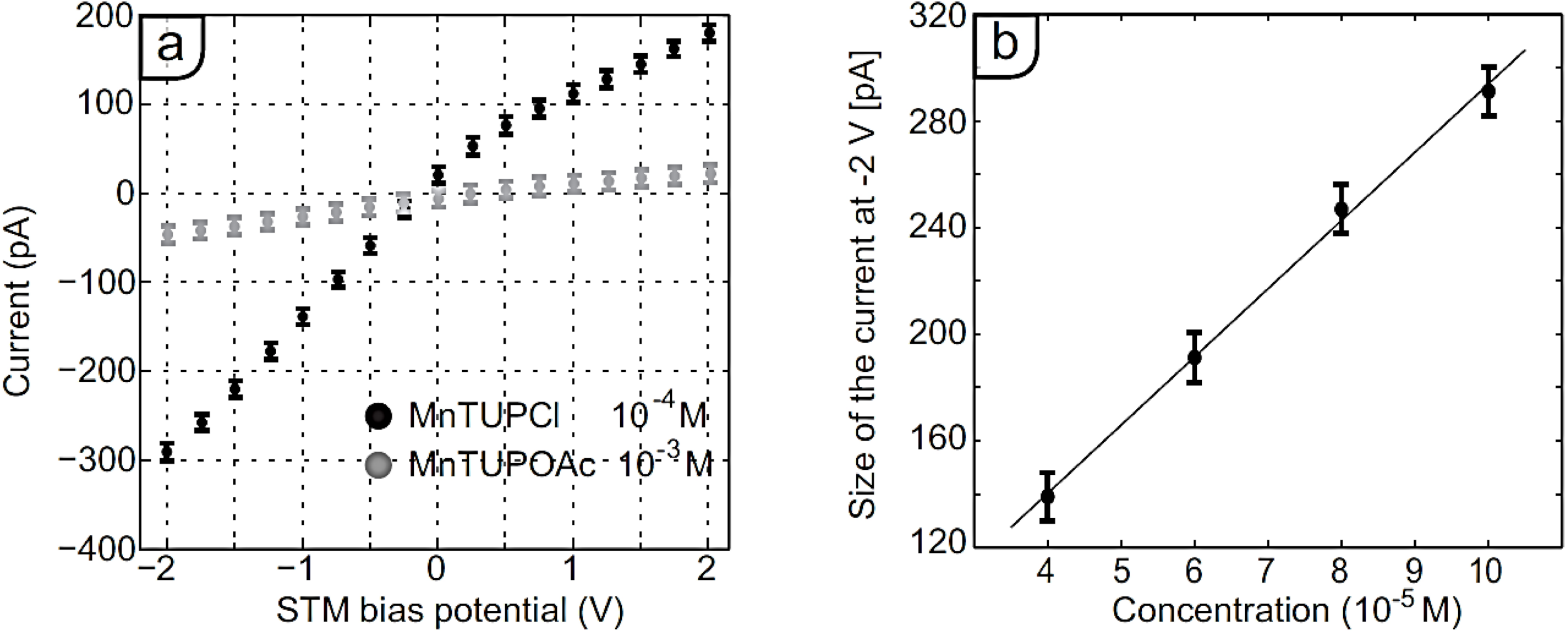
(a) Bias voltage dependence of the measured current in the STM setup at the interface of HOPG and a solution in 1-phenyloctane of **MnTUPCl** (*c* = 1 × 10^−4^ M, black) and of **MnTUPOAc** (*c* = 1 × 10^−3^ M, grey). (b) Concentration dependence of the measured current in the STM setup at a bias voltage of −2 V of **MnTUPCl** at the same interface (*c* = 1 × 10^−4^ M); the line is a linear fit through the data points. Both graphs show the average ±1 S.D. of 1000 measurements recorded within a time span of 1.5 min.

Next, the dependency of the observed current upon systematic variation of the bias voltage was monitored (black trace in Figure 2a). The observed dependency of the current on the concentration of the solutions at a set bias voltage of −2 V is plotted in Figure 2b. In a reference experiment under the same conditions, pure 1-phenyloctane displayed current changes of only 1–2 pA, independent of changes in bias voltage (not shown). This observation implies that it is not exclusively the solvent that is responsible for the current. The observed behavior can have different causes. For example, due to the polarization of the solvent and/or the solutes therein, a current may flow from the tip to the sample or vice versa. Such a polarization may be triggered by a voltage change. However, it can be expected that when the voltage after the change remains constant, after a certain time a new equilibrium will be reached in which the flow of charges has died out and the additional current decays to zero. In the case of the experiments with **MnTUPCl**, however, such a decay was never observed, and the current remained relatively stable as a function of the time. Alternatively, the current may be a result of the formation and presence of conducting assemblies of π-stacked porphyrins. However, in this case an exponential decay of the conductance would be expected with an increasing distance between the tip and sample [14], which again was not observed for solutions of **MnTUPCl**. The variations in the additional current did not increase when the tip–sample separation distance was varied between 5 and 20 µm (20 µm was the physical limit of the setup). Therefore, it is considered to be unlikely that the observed current is a result of conducting assemblies of π-stacked porphyrins connecting the tip with the HOPG surface.

The observed current may also be caused by reduction or oxidation reactions of the solutes at the tip and at the sample surface. Such reactions would result in so-called Faradaic currents between the tip and the sample. In the case of **MnTUPCl**, the following redox reactions at the tip or sample surface can be envisaged:

[1] $$2Mn(III)TUPCl+2{\text{e}}^{-}\to 2Mn(II)TUP+2{\text{Cl}}^{-}$$

[2] $$2{\text{Cl}}^{-}\to {\text{Cl}}_{2}+2{\text{e}}^{-}$$

We base these proposed reactions on the fact that the manganese center can be reduced from (III) to (II) [15–16], results from previous work that the substrates in an STM setup act as electrodes at which manganese porphyrins can be reduced [6–8], and the fact that also cobalt porphyrins can accept electrons from a HOPG surface in a liquid-STM setup [17–18]. While we have demonstrated that these redox reactions do not occur spontaneously in solutions of **MnTUPCl** in, e.g., a glass test tube [7], they do occur in the STM setup when two electrically connected conductive surfaces (tip and sample) are present. This suggests that adsorption of the molecules to these surfaces indeed activates their reactivity. Our proposal of redox reactions is supported by the observation that the magnitude of the current increases with an increasing concentration of **Mn(III)TUPCl** in the supernatant solution. Typically, Faradaic currents depend linearly on the concentration of the redox-active species, and Figure 2b indeed shows a linear dependence of the observed currents on the used concentration of **Mn(III)TUPCl**. Based on the proposed one-electron process, the measured currents of up to 300 pA agree with a reaction rate of 3 × 10^−15^ mol/s. The solutions applied to the surface contain about 3.5 × 10^−8^ mol of **MnTUPCl**, which is a sufficient amount to sustain the reactions for weeks, at least in principle. However, since we propose that the reduction of the manganese porphyrins takes place when they are adsorbed to the negatively biased electrode, their adsorption–desorption process must be dynamic. Once **Mn(II)TUP** complexes are formed, they must be able to desorb and replaced by molecules of **Mn(III)TUPCl** from the supernatant solution so that the reduction reactions can continue. Given the fact that the molecules of **MnTUPCl** are adsorbed to the interface via relatively weak physisorption interactions, such dynamics are likely.

In the proposed redox reactions, the chloride ligands, which are axially coordinated to the manganese centers, play an essential role in generating the observed currents. First, they dissociate when the manganese center is reduced. We propose that the ions are subsequently solvated by the 1-phenyloctane solvent and migrate to the other electrode (vide infra). Second, in a counter reaction they are oxidized to chlorine gas. No net current would be observed if one of the two processes would not occur. In order to check the proposed role of the chloride ions, an analogous porphyrin containing an acetate instead of a chloride axial ligand was synthesized (**MnTUPOAc**, Figure 1a). The acetate ligand cannot act as a reductor, and as a consequence a similar current as observed in the case of **MnTUPCl** should not occur. The currents measured upon the addition of 350 µL of a 1-phenyloctane solution of **MnTUPOAc** (*c* = 1 × 10^−3^ mM) to the modified STM setup are shown in gray in Figure 2a. Even at a ten times higher concentration of **MnTUPOAc** than that of **MnTUPCl** in the previously discussed experiments, the measured currents stayed between −50 ± 10 and +30 ± 10 pA upon variation of the bias voltage between −2 and +2 V, respectively. Similar results were obtained when a 350 µL 1-phenyloctane solution of **CuTUP** [10] (*c* = 1 × 10^−3^ mM), a closely related metal porphyrin without an axial ligand coordinated to the metal center, was used. Both observations support the proposed essential role of the chloride counterion for the redox reactivity of **MnTUPCl**.

To investigate the influence of the nature of the used electrodes, we varied the sample surfaces (HOPG and Au(111)) and the tip material (Pt_90_Ir_10_, Au and W). A summary of these experiments is given in Table 1. Additional currents were observed on both used surfaces (Au(111) and HOPG), and with all the used tip materials. The differences in observed currents for different systems demonstrate the complexity of characterizing redox reactions with a two-probe system (tip and sample) in a non-conducting liquid. Obviously, such a setup is not the perfect design for experiments to investigate redox behavior, but it is worthwhile to consider what its shortcomings are. It can be expected that the rates of the reactions in Equation 1 and Equation 2 depend, among other factors, on the surface at which the reactions occur [19]. This specific surface effect is highlighted by the observation that the magnitude of the observed currents is typically not mirrored when the sign of the applied bias voltage is switched (Table 1), indicating that when the two redox reactions occur at different sample electrodes this gives rise to different reaction rates. First, only the overall potential difference between the tip and the sample is known. The potential drops from the surfaces to the solution are governed by the material of the surface and structural properties of the interface, the type and polarizability of the solvent, the solutes (and their concentration), the presence of ions in the solvent, and a possible assembly of solutes at the interface. Furthermore, the reaction rates are determined by the chemical and electronic state of the sample surface, ion concentrations in the solution, and the availability of possible reaction intermediates. Most of these complications may be eliminated by adding a third, reference electrode to the setup and by using a conducting electrolyte (so that it turns into a so-called electrochemical (EC) STM [20–21]). At the same time, the absence of an electrolyte, which typically contains a high concentration of added salts, allows us to pinpoint the behavior of the axial ligand of our catalyst in the non-polar liquid in which also our catalysis studies were carried out. Still, our system shows several of the characteristics of a conventional electrochemical cell. Apart from the bias dependency of the reaction rate (both in terms of size and sign), also higher concentrations of the redox-active species lead to higher reaction rates, which points at increased dynamic exchange of the redox-active species at the sample and tip electrodes. Furthermore, the proposed transport of chloride ions through the 1-phenyloctane solvent basically makes it an electrolyte, albeit an unconventional one.

**Table 1:** Measured currents (in pA) between the tip and the sample in the modified liquid-STM setup as a function of the applied bias voltage (*V*_bias_). Porphyrin concentrations were 1 × 10^−4^ M, except for **MnTUPOAc** and **CuTUP** where a concentration of 1 × 10^−3^ M was used. Numbers between brackets indicate uncertainties (±1 S.D.).

			*V*_bias_		
	
substrate	solute	Tip	−2 V	0 V	2 V

solvent: 1-phenyloctane					

HOPG	**MnTUPCl**	PtIr	−1470 (130)	110 (17)	1710 (50)
HOPG	**MnTUPOAc**	PtIr	−50 (10)	−10 (10)	30 (5)
Au(111)	**MnTUPCl**	PtIr	−660 (80)	200 (20)	910 (30)
HOPG	**MnTUPCl**	Au	−770 (90)	230 (30)	1110 (40)
Au(111)	**MnTUPCl**	Au	−870 (90)	190 (30)	1190 (60)
HOPG	**MnTUPCl**	W	−700 (40)	40 (10)	700 (20)
HOPG	**CuTUP**	PtIr	−20 (3)	9.8 (5)	37 (2)
HOPG	none	PtIr	−7.8 (4)	−9.4 (4)	−9.6 (3)

solvent: *n*-tetradecane					

HOPG	**MnTUPCl**	PtIr	−11 (9)	−3 (9)	5 (9)
HOPG	none	PtIr	−10 (8)	−10 (9)	−10 (8)
Au(111)	**MnTUPCl**	PtIr	−7 (9)	−2 (8)	3 (9)
Au(111)	none	PtIr	−11 (8)	−11 (9)	−11 (8)

**Figure 3:**
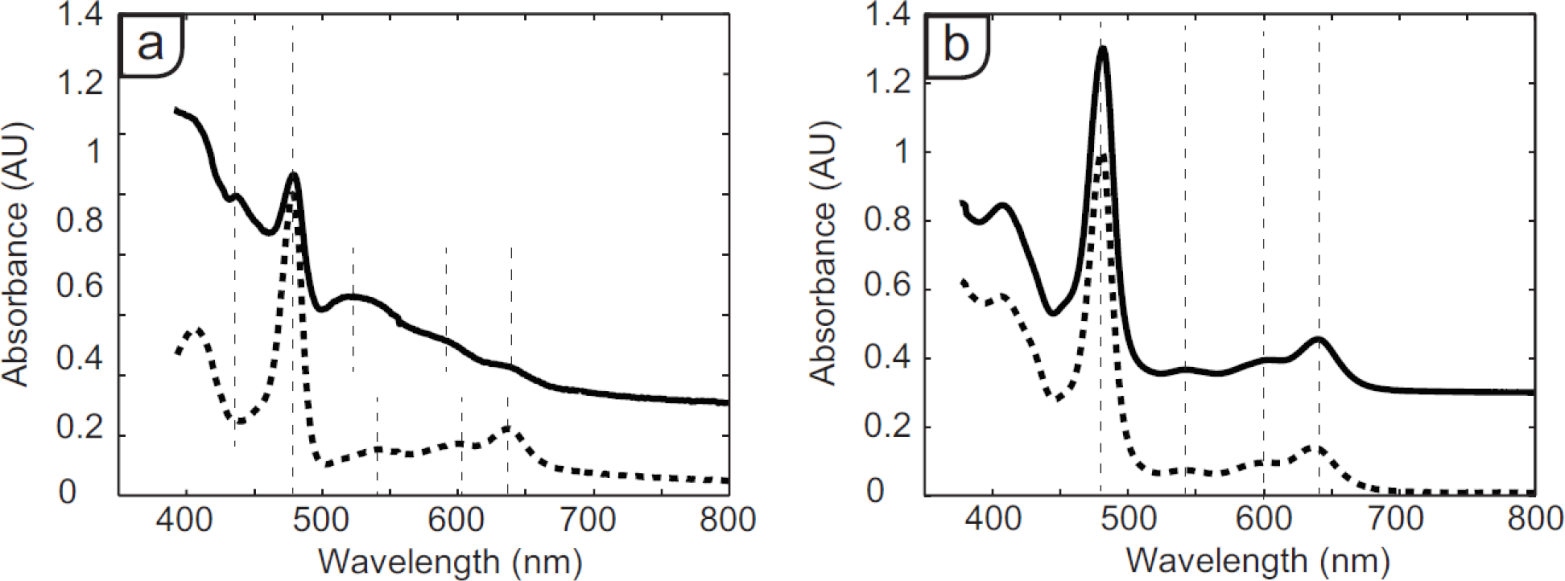
UV–vis spectra of sample (solid traces) and reference solutions (dashed traces of (a) **MnTUPCl** and (b) **MnTUPOAc** in 1-phenyloctane. The locations of main bands are indicated by narrow dashed vertical lines.

Assuming that the observed additional currents are caused by the proposed redox reactions (Equation 1 and Equation 2), **Mn(II)TUP** is generated, which may dissociate from the solid–liquid interface and dissolve in the supernatant solution. To investigate changes in supernatant composition during the STM experiments, ex situ UV–vis spectroscopy measurements were carried out. UV–vis spectra of supernatant solutions from different experiments in the modified STM setup using **MnTUPCl** and **MnTUPOAc** are depicted in Figure 3. The solid traces represent solutions that had been in the modified STM setup for four days, with the tip out of tunnel range and at a bias voltage of −2 V. The dashed traces (absorption value manually offset by 0.3 AU) are from reference solutions of the same composition, which were left to stand in an unsealed test tube next to the STM setup for the same four days. All used concentrations were 1 × 10^−4^ M.

The UV–vis spectra of the sample and reference solutions of **MnTUPOAc** (Figure 3b) are nearly identical, showing the characteristic strong Soret band at 481 nm and Q-bands at 604 and 640 nm. In contrast, the UV–vis spectra of the sample and reference solutions of **MnTUPCl** (Figure 3a) show that the chemical composition of the solution that was in the STM setup had clearly changed. Both spectra show a Soret band at 478 nm, but in the spectrum of the sample solution a small, new band has emerged at 437 nm. Also the first Q-band has blueshifted by 21 nm, and a larger background absorbance is observed. These spectral changes indicate that the molecules of **MnTUPCl** are reactive in the STM setup under the influence of the bias voltage.

Following our hypotheses described above, the solution might also contain molecules of **Mn(II)TUP**. A blue shift of the Soret band from 478 to 437 nm is indeed in line with a one-electron reduction of the porphyrin Mn(III) center to Mn(II) [22–23]. However, since this species is highly reactive to oxidation by air, other changes in the UV–vis spectrum may additionally be caused by the formation of oxidation products of **Mn(II)TUP**. Because the spectrum is quite broad and no clear other bands can be recognized, it is at this stage difficult to draw conclusions about the identity of these products.

The occurrence of the additional currents appeared to depend on the nature and polarity of the solvent. When a solution of **MnTUPCl** in the non-aromatic and less polar solvent *n*-tetradecane was subjected to a bias voltage in the modified STM setup, no significant additional currents were observed compared to reference experiments in which the porphyrins were absent (Table 1). This does not directly imply that the redox reactions in Equation 1 and Equation 2 do not occur under these conditions; the redox reaction rates may be very low, i.e., below 2.5 × 10^7^ e^−^/s (comparable with a current of approx. 4 pA), and not exceed the noise level of the STM setup. Possible aspects that could cause the difference in observed current between solutions of **MnTUPCl** in 1-phenyloctane and *n*-tetradecane are: (i) a better solubility of chloride ions and/or **Mn(II)TUP** species in 1-phenyloctane compared to *n*-tetradecane, and (ii) a difference in dielectric constants of the used solvents, which are 2.26 and 2.03, respectively. A different dielectric constant may lead to a different potential decay at the interfaces, which in turn may influence the reaction rates. However, since the difference in dielectric constant between the two solvents is small, we propose the first explanation as being more likely. An enhanced solubility of the ions in the aromatic solvent 1-phenyloctane may be the result of attractive anion–π interactions [24].

## Conclusion

**MnTUPCl** dissolved in 1-phenyloctane and placed in a tunnel junction yields high additional currents. We attribute them to Faradaic currents and put forward two possible explanations for the reduction of the manganese centers of the molecules of **MnTUPCl** at the Au(111)–*n*-tetradecane interface in a liquid-STM, i.e., the coordination of a gold atom from the top-most gold layer to **MnTUPCl** in an axial ligand-like fashion, or an active reduction as the result of the donation of an electron from the gold surface to the **MnTUPCl**. In this work it was shown that the use of the aromatic solvent 1-phenyloctane was accompanied by the emergence of large additional currents in the STM setup. These currents are in line with the occurrence of redox reactions at the two electrodes (surface and tip), in which chloride axial ligands dissociate upon reduction of the manganese centers at one electrode, after which the chloride ions are oxidized to chlorine gas at the other electrode. These reactions are supported by several observations: (i) The observed additional currents are stable as a function of the time and depend linearly on the applied **MnTUPCl** concentration in the supernatant solution, which would be expected for the proposed redox reactivity; (ii) when one of the redox partners was eliminated, i.e., by replacing the chloride axial ligand of the Mn(III) porphyrin by a redox-inert acetate ligand (**MnTUPOAc**), no such additional currents are observed; (iii) ex situ UV–vis spectra of the supernatant solution significantly changed over time, indicating reactivity in the modified STM setup and the formation of Mn(II) porphyrin, which is one of the expected products of the proposed redox reactions.

Since apparently the axial ligand of Mn(III) porphyrins can play an essential role in the redox behavior of these compounds at a solid–liquid interface in a liquid-STM setup, our future research will be focused on the variation of these ligands and their role for the use of the metal porphyrins in catalysis. In particular, we intend to study with liquid-STM the catalytic properties of **MnTUPOAc** in the epoxidation of alkenes, and compare its performance to that of **MnTUPCl** under the same conditions.

## Experimental

### Materials and methods

All commercially obtained chemicals were used without further purification unless stated otherwise. The purity of 1-phenyloctane, the solvent in which the additional currents were observed, was confirmed by ^1^H NMR spectroscopy; moreover, the currents were observed in multiple batches of this solvent, of two different suppliers (ACROS Organics or Sigma-Aldrich), independent whether it was vacuum-distilled prior to use or used as received. For TLC analysis, TLC silicagel 60 F254 (Merck, Burlington, MA, USA) and for column chromatography silica gel 0.035–0.070 mm, (Acros, Branchburg, N.J., USA) was used. MALDI-TOF mass spectra were measured in reflective mode with dithranol as a matrix on a Bruker Microflex LRF MALDI-TOF mass spectrometer (Bruker, Billerica, MA, USA). UV–vis spectra were recorded on a Varian Cary 50 UV–vis spectrophotometer.

### Syntheses

#### Synthesis of MnTUPOCl and CuTUP

Compounds **MnTUPCl** [7] and **CuTUP** [10] were synthesized according to literature procedures.

#### Synthesis of MnTUPOAc

A solution of the free ligand **TUP** [7] (50 mg, 0.054 mmol) and Mn(OAc)_2_·4H_2_O (52 mg, 0.21 mmol) in argon-purged DMF (5 mL) was stirred and heated at reflux under argon for 3 h. After cooling, the solvent was evaporated and the residue was re-dissolved in CH_2_Cl_2_ (20 mL). The solution was extracted with water (2 × 50 mL) and then concentrated to a volume of 5 mL. An equal volume of a saturated aqueous NaOAc solution was added and the mixture was vigorously stirred open to air for 16 h. The product in the organic layer was subsequently purified by column chromatography (silica 60H, eluent CH_2_Cl_2_/MeOH 9:1, v/v) to yield **MnTUPOAc** (48 mg, 86%) as a dark green solid. MALDI-TOFMS *m*/*z* 979 [M‒OAc]^+^; UV–vis (CH_2_Cl_2_) λ/nm (log(ε/M^−1^cm^−1^)): 397 (4.52), 375 (4.60), 481 (4.95), 548 (3.35), 604 (3.56), 640 (4.01).

### Experiments in the liquid-STM setup

A solution of a porphyrin compound was dissolved in an appropriate solvent (see text) and applied to a freshly cleaved HOPG substrate (10 × 10 mm^2^, NT-MDT, ZYB) or Au(111) film (10 × 10 mm^2^ with a thickness of 200 nm evaporated on freshly cleaved muscovite mica) which was mounted into a liquid-cell in a custom-built liquid-STM setup [7]. The STM-tip (mechanically cut) was immersed in a typical volume of 350 µL of the solution. The used concentrations of porphyrin varied between 1 × 10^−4^ and 1 × 10^−3^ M and are mentioned at the relevant experiments in the main text. The STM measurements were performed in constant-current mode using an Omicron Scala SPM controller. All experiments were performed in the thermostatted environment (21.5 ± 0.5 °C) of the NanoLab Nijmegen.
